# Pharmacologic targeting of drug-induced enhancers

**DOI:** 10.18632/oncoscience.354

**Published:** 2017-06-22

**Authors:** Jon S. Zawistowski, Samantha M. Bevill, Gary L. Johnson

**Affiliations:** Department of Pharmacology, Lineberger Comprehensive Cancer Center, University of North Carolina School of Medicine, Chapel Hill, NC 27599, USA

**Keywords:** epigenomic landscape, adaptive transcriptional response, MEK inhibition, P-TEFb, enhancer remodeling

In contrast to acquired resistance to targeted therapeutics via genomic changes, adaptive resistance in cancer involves rapid cellular reprogramming at a non-genomic level. In triple negative breast cancer (TNBC) we have recently described robust transcriptional adaptation to MEK inhibition (MEKi) by the FDA-approved MEK inhibitor, trametinib, in both TNBC patients and preclinical models [1]. This transcriptional adaptation includes upregulation of bypass signaling molecules including receptor tyrosine kinases (RTKs) to reactivate cellular proliferation programs in the presence of the initial drug challenge [2].

Rather than attempting to combat RTK upregulation using a secondary kinase inhibitor in conjunction with the initial, primary targeted therapeutic, we have employed a strategy to broadly inhibit MEKi-induced transcriptional adaptation through the use of BET bromodomain inhibitors. BET protein family bromodomains bind to acetylated lysine moieties of histone subunits or transcription factors to regulate transcriptional elongation via recruitment of P-TEFb, a RNA polymerase II regulatory complex containing CDK9 and Cyclin T1. In TNBC we found the combination of MEKi + BET bromodomain inhibition (BETi) resulted in durable tumor cell growth inhibition not obtained with trametinib alone, nor that obtained with different kinase inhibitor combinations [3]. Importantly, we observed *in vivo* MEKi + BETi synergy in orthotopic SUM159PT xenograft and T11 and 2225 mouse orthotopic syngeneic transplant models of TNBC. BETi synergism with targeted kinase inhibitors is not limited to TNBC, as we have shown that lapatinib + BETi results in durable growth suppression in HER2+ breast cancer [3].

A central question is the mechanism of BETi efficacy in the context of suppressing adaptation to targeted therapeutics. Seminal studies have implicated the BET family bromodomain protein BRD4 in enhancer and super-enhancer mediated control of developmentally regulated genes [4, 5]. Furthermore, evidence for BRD4 function at stimulus-dependent, inducible enhancers has been accumulating in the context of γ-secretase inhibitors in T-cell acute lymphoblastic leukemia [6] and following tumor necrosis factor alpha (TNFα) treatment in primary human umbilical vein endothelial cells [7]. We asked if BRD4 function at enhancers is similarly critical for adaptive transcription in response to MEK1/2 inhibition by trametinib.

We found vast chromatin remodeling in the form of *de novo* enhancer formation and remodeling in response to trametinib in TNBC cells. Enhancers with pronounced BRD4 density and co-occupied with prototypical enhancer marks (H3K27ac, MED1, H3K4me1) were rapidly (1-4 h) formed genome-wide, including at sites proximal to receptor tyrosine kinase loci including *DDR1, KDR, FGFR2* and *PDGFRB*, each influential in TNBC adaptive resistance. Trametinib-responsive enhancers were remodeled across the genome but with BETi the total number of enhancers remained near baseline. In fact, BETi was able to disrupt enhancers seeded in response to trametinib. We observed correlation of transcriptional induction of genes proximal to the enhancer density induced by trametinib and a corresponding correlative decrease in transcript levels of the cognate genes of the seeded enhancers with combination MEKi+BETi treatment.

**Figure 1:**
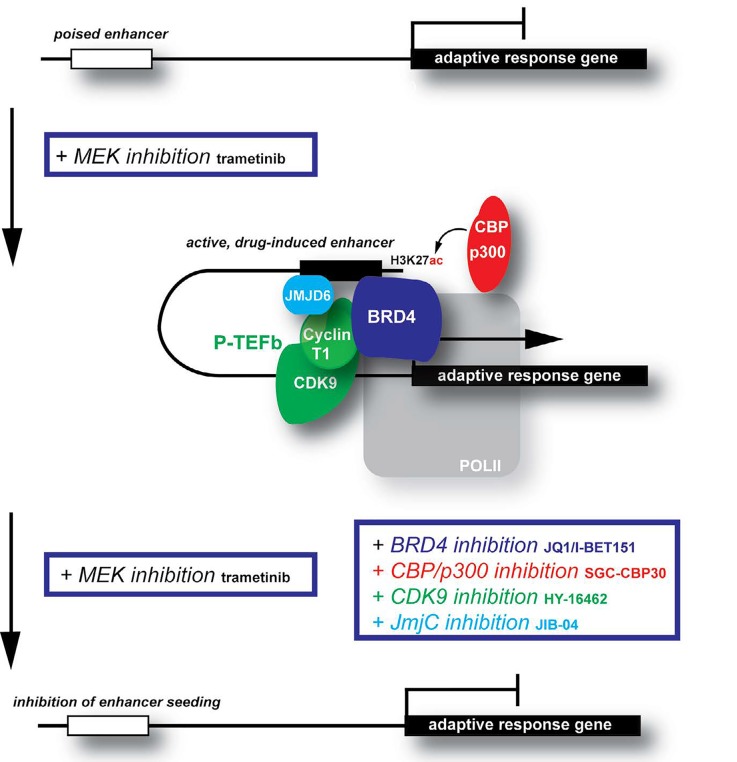
Enhancer targeting with epigenetic inhibitors as a strategy to attenuate adaptive transcription to MEK inhibition.

Does the enhancer paradigm provide a potential source of pharmacologic targets for attenuating adaptive transcription beyond that of BRD4? In addition to BETi, we found that pharmacological perturbation of CBP/p300 acetyltransferase, capable of depositing acetylation at H3K27 of enhancers, or core P-TEFb constituent CDK9 abrogated adaptive RTK upregulation elicited by MEKi (Figure 1). BRD4 directly associates with the transcriptional regulator JMJD6, a JmjC family demethylase [8] and, accordingly, the use of a pan-JmjC family demethylase inhibitor or siRNA targeting JMJD6 diminished RTK adaption. Similar response abrogation was achieved with depletion of the histone H3 lysine 36 methyltransferase NSD3 [8] or by depleting CDK7, whose transcriptional regulatory activity is conferred by phosphorylation of its substrates including CDK9 and the carboxy-terminal domain (CTD) of RNA polymerase II. Additionally, as evidence for functional roles of enhancer (eRNA) transcription in transcriptional regulation of coding genes continues to mount, it is likely that eRNA-associated factors or the eRNA molecules themselves will be in the realm of targets to consider for epigenetic strategies to prevent adaptive transcription. While small molecule inhibitors are currently not yet available or are pre-Phase I for many of these purported strategies to block adaptive resistance, our preclinical data suggest that protein complexes of drug-induced enhancers may be a largely untapped frontier of epigenetic targets to block adaptive bypass resistance to targeted therapies.
